# Predictors of Adherence to Stroke Prevention in the BALKAN-AF Study: A Machine-Learning Approach

**DOI:** 10.1055/s-0042-1755617

**Published:** 2022-09-23

**Authors:** Monika Kozieł-Siołkowska, Sebastian Siołkowski, Miroslav Mihajlovic, Gregory Y.H. Lip, Tatjana S. Potpara, on behalf of the BALKAN-AF Investigators

**Affiliations:** 1 Liverpool Centre for Cardiovascular Science, University of Liverpool and Liverpool Heart & Chest Hospital, Liverpool, United Kingdom; 2 1st Department of Cardiology and Angiology, Silesian Centre for Heart Diseases, Zabrze, Poland; 3 Cardiology Clinic, Clinical Centre of Serbia, Belgrade, Serbia; 4 School of Medicine, Belgrade University, Belgrade, Serbia; 5 Department of Clinical Medicine, Aalborg University, Aalborg, Denmark

**Keywords:** ABC pathway, atrial fibrillation, BALKAN-AF study, oral anticoagulants, stroke prevention

## Abstract

**Background** Compared with usual care, guideline-adherent stroke prevention strategy, based on the ABC (Atrial fibrillation Better Care) pathway, is associated with better outcomes. Given that stroke prevention is central to atrial fibrillation (AF) management, improved efforts to determining predictors of adherence with ‘A’ (avoid stroke) component of the ABC pathway are needed.

**Purpose** We tested the hypothesis that more sophisticated methodology using machine learning (ML) algorithms could do this.

**Methods** In this post-hoc analysis of the BALKAN-AF dataset, ML algorithms and logistic regression were tested. The feature selection process identified a subset of variables that were most relevant for creating the model. Adherence with the ‘A’ criterion of the ABC pathway was defined as the use of oral anticoagulants (OAC) in patients with AF with a CHA _2_ DS _2_ -VASc score of 0 (male) or 1 (female).

**Results** Among 2,712 enrolled patients, complete data on ‘A’-adherent management were available in 2,671 individuals (mean age 66.0 ± 12.8; 44.5% female). Based on ML algorithms, independent predictors of ‘A-criterion adherent management’ were paroxysmal AF, center in capital city, and first-diagnosed AF. Hypertrophic cardiomyopathy, chronic kidney disease with chronic dialysis, and sleep apnea were independently associated with a lower likelihood of ‘A’-criterion adherent management.

ML evaluated predictors of adherence with the ‘A’ criterion of the ABC pathway derived an area under the receiver-operator curve of 0.710 (95%CI 0.67–0.75) for random forest with fine tuning.

**Conclusions** Machine learning identified paroxysmal AF, treatment center in the capital city, and first-diagnosed AF as predictors of adherence to the A pathway; and hypertrophic cardiomyopathy, chronic kidney disease with chronic dialysis, and sleep apnea as predictors of non adherence.

## Introduction

Atrial fibrillation (AF) constitutes significant burden to physicians, patients, and healthcare systems globally. 1 The complexity of AF requires an integrated or holistic approach to its management, by careful characterization and evaluation, 2 followed by implementation of Atrial fibrillation Better Care (ABC) pathway (ie. ‘A’ Avoid stroke; ‘B’ Better symptom management; ‘C’ Cardiovascular and Comorbidity optimization). 3 The ABC pathway simplifies integrated care of AF patients among different specialties, and across all healthcare levels. Compared with usual care, guideline-adherent stroke prevention strategy, based on the ABC pathway, is associated with better clinical outcomes. 4 5

The risk of stroke is increased 5-fold in patients with AF, but this risk is not homogeneous and is dependent upon the presence of stroke risk factors. Given that stroke prevention is central to AF management, 3 improve efforts in determining predictors of adherence with ‘A’ (avoid stroke) component are needed.

Guideline-adherent management is associated with better outcomes. 6 7 However, adherence to the guidelines in real-world clinical practice might be suboptimal for many reasons. 4 Importantly, registries have provided significant evidence on adherence to guidelines, and contemporary AF management, especially for the under-represented patient groups.

In this ‘proof of concept’ study, we tested the hypothesis that more sophisticated methodology using machine learning (ML) algorithms could potentially improve our determination of predictors of adherence with ‘A’ (avoid stroke) component of the ABC pathway. We compared ML to traditional methods of predicting the adherence to stroke prevention. Moreover, we presented data on the use of various ML methods. Such an approach is increasingly used in AF research to improve detection, risk analysis and improve AF management. 8

## Methods

A detailed description of the BALKAN-AF study has been previously published. 9 This 14-week multicentre ‘snapshot’ registry of consecutive patients with electrocardiographically confirmed AF was designed and conducted prospectively by the Serbian Atrial Fibrillation Association (SAFA). Enrolment to the study lasted from December 2014 to February 2015 in collaboration with the National Cardiology Associations and Societies or Working Groups in seven Balkan countries (Albania, Bosnia & Herzegovina, Bulgaria, Croatia, Montenegro, Romania, and Serbia). A total of 49 centres of academic and non-university hospitals and outpatient health centres were sites in the BALKAN-AF study. The sites were chosen by the respective National Coordinator. The registry was approved by the national / local institutional review board in participating countries. The study has obtained an ethical approval.

Patients aged < 18 years or those with prosthetic mechanical heart valves or valvular heart disease with indications for surgical repair were exclusion criteria for this study. A signed patient informed consent form was required during enrolment. The study protocol is concordant with the Declaration of Helsinki.

Data were collected and stored specifically for the study using an electronic case report form (eCRF). Patient signs or symptoms, characteristics and healthcare setting, diagnostic procedures performed within the last 12 months and during enrolment, and AF management at enrolment and at discharge were included in the eCRFs. Stroke risk was evaluated using the CHA _2_ DS _2_ -VASc score. 10 Bleeding risk was assessed using the HAS-BLED [hypertension, abnormal renal /liver function, stroke, bleeding history or predisposition, labile International Normalised Ratio (INR), elderly (>65 years), drugs or alcohol concomitantly] score. 11 The cardiovascular risk factors, risk scores and diseases definitions were defined using individual European Society of Cardiology guidelines, other guidelines, scientific statements and textbooks described previously in supplementary information. 12

Regular monitoring of centres and follow-up visits were not performed due to relatively short period of the study. National investigators and coordinators were in charge of verification of the consecutiveness of enrolled patients and correctness and completeness of entered data.

Adherence with the ‘A’ criterion of the ABC pathway was defined as the use of oral anticoagulants (OAC) in patients with AF with a CHA _2_ DS _2_ -VASc score of 0 (male) or 1 (female).

### Statistical Analysis

Categorical variables were presented as absolute frequencies and percentages. Between-group comparisons were shown with Student's *t* -test or Mann-Whitney test. The descriptive analysis involved baseline characteristics of patients. The comparative analyses associated with ‘A-adherent management’ were previously published. 13 They were calculated using univariate and multivariate logistic regression analyses. Statistically significant variables in univariate logistic regression model were included into multivariate logistic regression model to identify multivariable predictors of the use of ‘A-adherent management’. Results were shown as odds ratio (OR) with 95% confidence interval (CI). A two-sided *p* value of less than 0.05 was qualified as statistically significant. Analyses were calculated using SAS software version 9.4 (SAS Institute, Inc., Cary, NC, USA).

### Machine Learning

**Fig. 1:**
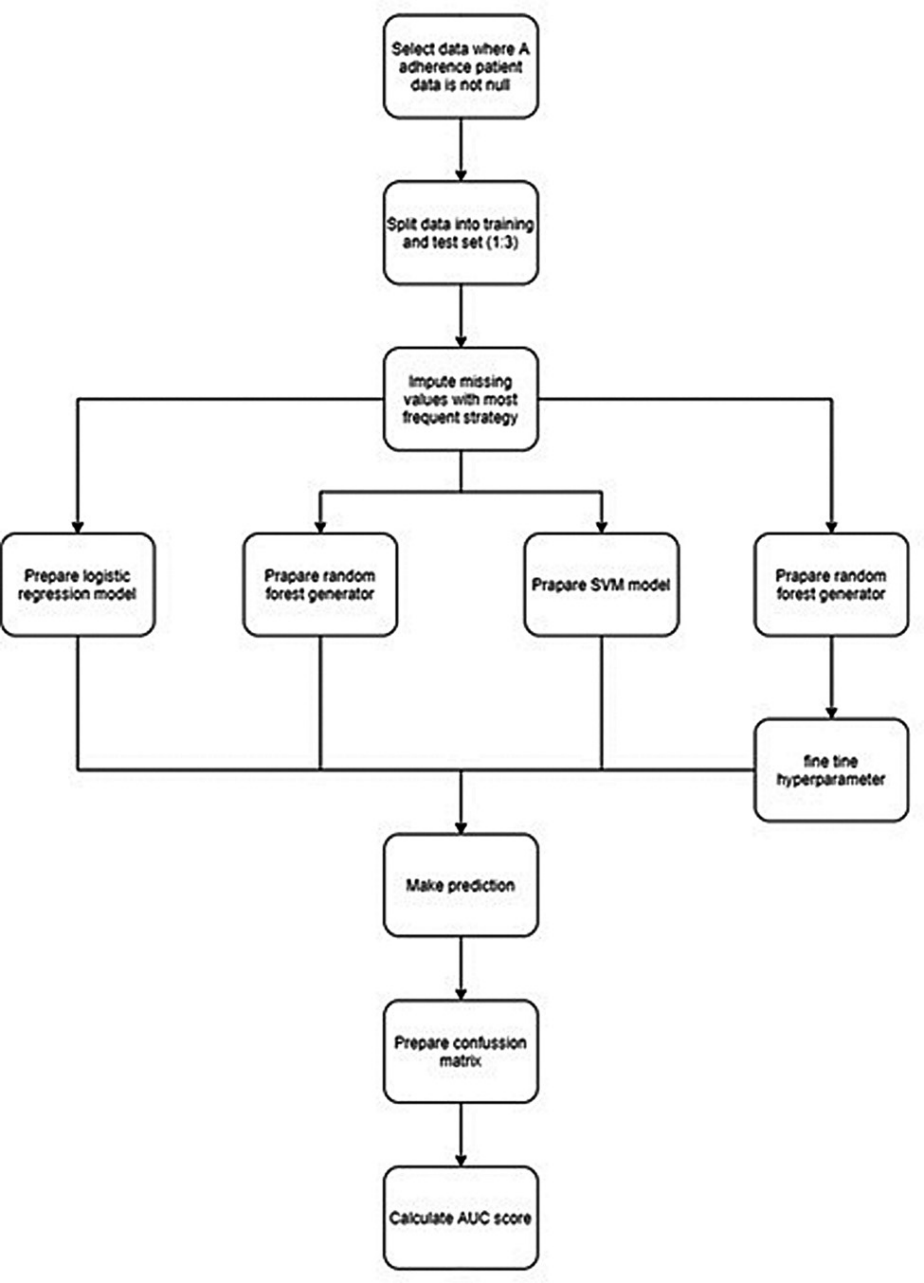
**Logistic regression and machine learning methods. Abbreviation:** AUC; area under the receiver-operator curve

The main strategy that was used to calculate and compare different ML methods is presented in Fig. 1 . Three ML models were tested: (i) support vector machines with linear Kernel model; (ii) random forest model without fine tuning model; and (iii) random forest model after fine tuning model. Each model was trained on a training set (75% of the data) and evaluated on test set (25% of data). Every model was calculated separately, then comments were made after checking predictions.

Every step of calculations were prepared in Python language. Preparing the data, we were using only the cases where strategy ‘A-criterion adherent management’ was non-empty. Missing data for independent variable columns were imputed using the most frequent method by ‘SimpleImputer’ from ‘sklearn’ library. Variables were scaled using ‘StandardScaler’ from ‘sklearn’ library. Random forest generator (without fine tuning) was prepared with n_estimator equals 10. Then, we fine tuned this model with n estimators in range 10 to 2000 with 10 values jumps. The best random forest generator was prepared with 1115 n_estimators. Support Vector Machine model was prepared with linear Kernel model.

## Results

In this post-hoc analysis, 2712 patients were enrolled, and complete data on adherence with the ‘A’ criterion of the ABC pathway were available in 2671 patients (98.5%; mean age 66.0 ± 12.8 years; 44.5% female). Patient characteristics of the study population are shown in Table 1 .

**Table 1:** Patient baseline characteristics

Variable	‘A-adherent management’ n = 1965	‘A-non-adherent management’ n = 706	*p* -value
Age, years (mean ± SD)	69.0 ± 10.2	69.6 ± 12.5	0.191
Age 65–74 years (%)	663 (33.7)	215 (30.5)	0.111
	665 (33.8)	278 (39.4)	0.008
Female sex (%)	874 (44.5)	314 (44.5)	0.973
Alcohol abuse (%)	80 (4.1)	30 (4.2)	0.838
Body mass index (mean ± SD)	28.0 ± 4.4	26.9 ± 4.1	<0.001
First diagnosed AF (%)	376 (19.1)	251 (35.6)	<0.001
Paroxysmal AF (%)	591 (30.1)	366 (51.8)	<0.001
Persistent AF (%)	312 (15.9)	71 (10.1)	<0.001
Permanent AF (%)	865 (44.0)	221 (31.3)	<0.001
Arterial hypertension (%)	1607 (81.8)	508 (72.0)	<0.001
Heart failure ever (%)	875 (44.5)	286 (40.5)	0.071
Coronary artery disease (%)	571 (29.1)	249 (35.3)	<0.001
Prior PCI/ stenting (%)	163 (8.1)	62 (8.8)	0.282
Prior myocardial infarction (%)	249 (12.7)	119 (16.9)	0.248
Mitral valve disease (%)	654 (33.3)	191 (27.1)	0.002
Aortic valve disease (%)	213 (10.8)	87 (12.3)	0.285
Dilated cardiomyopathy (%)	183 (9.3)	33 (4.7)	<0.001
Hypertrophic cardiomyopathy (%)	41 (2.1)	12 (1.7)	0.528
Peripheral artery disease (%)	93 (4.7)	29 (4.1)	0.495
Diabetes mellitus	489 (24.9)	179 (25.4)	0.776
Chronic kidney disease	309 (15.7)	102 (14.4)	0.420
Chronic hepatic disease	63 (3.2)	33 (4.7)	0.069
Prior stroke	209 (10.6)	71 (10.1)	0.674
Prior TIA	57 (2.9)	26 (3.7)	0.301
Prior bleeding	93 (4.7)	39 (5.5)	0.403
CHA _2_ DS _2_ -VASc score	3.4 ± 1.8	3.4 ± 2.0	0.661
HAS-BLED score	2.0 ± 1.2	1.8 ± 1.2	<0.001

Abbreviations: AF, atrial fibrillation; CHA _2_ DS _2_ -VASc; HAS-BLED, hypertension, abnormal renal /liver function, stroke, bleeding history or predisposition, labile International Normalised Ratio (INR), elderly (>65 years), drugs or alcohol concomitantly; PCI, percutaneous coronary interventions; SD, standard deviation; TIA, transient ischemic attack.

A simple logistic regression model for predictors of adherence with the ‘A’ criterion of the ABC pathway in the BALKAN region has been previously published 13 ; this showed that capital city, hypertension, dilated cardiomyopathy, thyroid disease, and treatment by cardiologist were independent predictors of adherence with ‘A’ criterion of the ABC pathway. Age ≥80 years, paroxysmal AF, and coronary artery disease were predictors of decreased adherence with ‘A’ criterion. 13

### Machine Learning

According to *support vector machines with linear Kernel model* , independent predictors of ‘A-criterion adherent management’ were as follows: CHA _2_ DS _2_ -VASc score, capital city, HAS-BLED score, CHA _2_ DS _2_ -VASc score ≥2, and mitral valve disease, Table 2 .

**Table 2:** Support vector machines with linear Kernel model for predictors of ‘A- criterion adherent management’

Support vector machines	
Feature value	Feature name
0.000242319873	score_CHA2DS2_VASc
0.000050269139	Capital_city
0.000048868996	score_HASBLED
0.000025492900	CHA2DS2VASc_of_2_or_more
0.000025182634	Mitral_valve_disease
0.000013552897	Valvular_disease
0.000011264013	Hospital_based_health_centre
0.000009945373	StableCAD
0.000009607100	Cardiologist
0.000005418319	PRIOR_MI_yes
0.000000877073	SLEEP_APNEA_yes
−0.000007977473	HCM
−0.000008169722	CAD_YES_CABG_yes
−0.000010532709	DCM
−0.000011218656	Aortic_valve_disease
−0.000012548092	Asymptomatic_currently
−0.000012768802	CAD_yes
−0.000013038458	PRIOR_TIA_yes
−0.000014002261	COPD_yes
−0.000014773701	Permanent_AF
−0.000016551256	HASBLED_of_3_or_more
−0.000023720672	University_centre
−0.000024071528	BLEEDING_EVENTS_yes
−0.000025166427	THYROID_DISEASE_yes
−0.000028964017	CKD_yes
−0.000030826819	PAD_yes
−0.000032045520	Age_80
−0.000036075120	PCI_Stent_yes
−0.000037642888	MALIGNANCY_yes
−0.000038990629	FIRST_DIAG_AF_yes
−0.000045796416	Vascular_disease
−0.000048134763	AF_Paroxysmal
−0.000052335890	HF_for_CHA2DS2VASc
−0.000056998545	HTN_yes
−0.000061853055	Female_gender
−0.000076459485	PRIOR_STROKE_yes
−0.000080565286	DIABETES_yes
−0.000129699179	Age_65_74
−0.000206825992	Age_75
−0.126120021028	CKD_CHRONIC_DIALYSIS_yes

Abbreviations: AF, atrial fibrillation; CABG, coronary artery bypass grafting; CAD, coronary artery disease; CHA _2_ DS _2_ -VASc; HAS-BLED, hypertension, abnormal renal /liver function, stroke, bleeding history or predisposition, labile International Normalised Ratio (INR), elderly (>65 years), drugs or alcohol concomitantly; HCM, hypertrophic cardiomyopathy; HF, heart failure; HTN, hypertension; MI, myocardial infarction; PAD, peripheral artery disease; PCI, percutaneous coronary intervention, TIA; transient ischemic attack.

According to the random forest model without fine tuning model, independent predictors of ‘A-criterion adherent management’ were: CHA _2_ DS _2_ -VASc score, HAS-BLED score, capital city, female gender, and first-diagnosed AF, Table 3 .

**Table 3:** Random forest model without fine tuning for predictors of the use of ‘A-criterion adherent management’

Random Forest Generator without fine tuning	
Feature value	Feature name
0.1	score_CHA2DS2_VASc
0.08	score_HASBLED
0.05	Capital_city
0.04	Female_gender
0.04	FIRST_DIAG_AF_yes
0.04	AF_Paroxysmal
0.03	University_centre
0.03	Cardiologist
0.03	Age_65_74
0.03	Age_80
0.03	Asymptomatic_currently
0.03	HTN_yes
0.03	DIABETES_yes
0.03	COPD_yes
0.03	HF_for_CHA2DS2VASc
0.02	Hospital_based_health_centre
0.02	Age_75
0.02	CAD_yes
0.02	Mitral_valve_disease
0.02	Aortic_valve_disease
0.02	CKD_yes
0.02	THYROID_DISEASE_yes
0.02	PRIOR_STROKE_yes
0.02	BLEEDING_EVENTS_yes
0.02	HASBLED_of_3_or_more
0.02	StableCAD
0.02	Vascular_disease
0.02	Permanent_AF
0.02	Valvular_disease
0.01	PRIOR_MI_yes
0.01	PCI_Stent_yes
0.01	CAD_YES_CABG_yes
0.01	DCM
0.01	HCM
0.01	PAD_yes
0.01	CKD_CHRONIC_DIALYSIS_yes
0.01	MALIGNANCY_yes
0.01	PRIOR_TIA_yes
0.01	CHA2DS2VASc_of_2_or_more
0	SLEEP_APNEA_yes

Abbreviations: AF, atrial fibrillation; CABG, coronary artery bypass grafting; CAD, coronary artery disease; CHA _2_ DS _2_ -VASc; HAS-BLED, hypertension, abnormal renal /liver function, stroke, bleeding history or predisposition, labile International Normalised Ratio (INR), elderly (>65 years), drugs or alcohol concomitantly; HCM, hypertrophic cardiomyopathy; HF, heart failure; HTN, hypertension; MI, myocardial infarction; PAD, peripheral artery disease; PCI, percutaneous coronary intervention; TIA, transient ischemic attack.

According to the random forest model after fine tuning model, independent predictors of ‘A-adherent management’ were: CHA _2_ DS _2_ -VASc score, paroxysmal AF, capital city, HAS-BLED score, and first-diagnosed AF, Table 4 .

**Table 4:** Random forest model after fine tuning for predictors of ‘A-criterion adherent management’

Random Forest Generator with fine tuning	
Feature value	Feature name
0.1	score_CHA2DS2_VASc
0.08	AF_Paroxysmal
0.07	Capital_city
0.06	score_HASBLED
0.05	FIRST_DIAG_AF_yes
0.04	HTN_yes
0.03	University_centre
0.03	Cardiologist
0.03	Age_80
0.03	Female_gender
0.03	COPD_yes
0.02	Hospital_based_health_centre
0.02	Age_75
0.02	Asymptomatic_currently
0.02	CAD_yes
0.02	PRIOR_MI_yes
0.02	DCM
0.02	Mitral_valve_disease
0.02	DIABETES_yes
0.02	CKD_yes
0.02	MALIGNANCY_yes
0.02	THYROID_DISEASE_yes
0.02	PRIOR_STROKE_yes
0.02	HASBLED_of_3_or_more
0.02	StableCAD
0.02	HF_for_CHA2DS2VASc
0.02	Vascular_disease
0.02	Permanent_AF
0.02	Valvular_disease
0.01	Age_65_74
0.01	PCI_Stent_yes
0.01	CAD_YES_CABG_yes
0.01	HCM
0.01	Aortic_valve_disease
0.01	PAD_yes
0.01	CKD_CHRONIC_DIALYSIS_yes
0.01	SLEEP_APNEA_yes
0.01	PRIOR_TIA_yes
0.01	BLEEDING_EVENTS_yes
0.01	CHA2DS2VASc_of_2_or_more

Abbreviations: AF, atrial fibrillation; CABG, coronary artery bypass grafting; CAD, coronary artery disease; CHA _2_ DS _2_ -VASc; HAS-BLED, hypertension, abnormal renal /liver function, stroke, bleeding history or predisposition, labile International Normalised Ratio (INR), elderly (>65 years), drugs or alcohol concomitantly; HCM, hypertrophic cardiomyopathy; HF, heart failure, HTN, hypertension, MI, myocardial infarction; PAD, peripheral artery disease; PCI, percutaneous coronary intervention; TIA, transient ischemic attack.

**Fig. 2:**
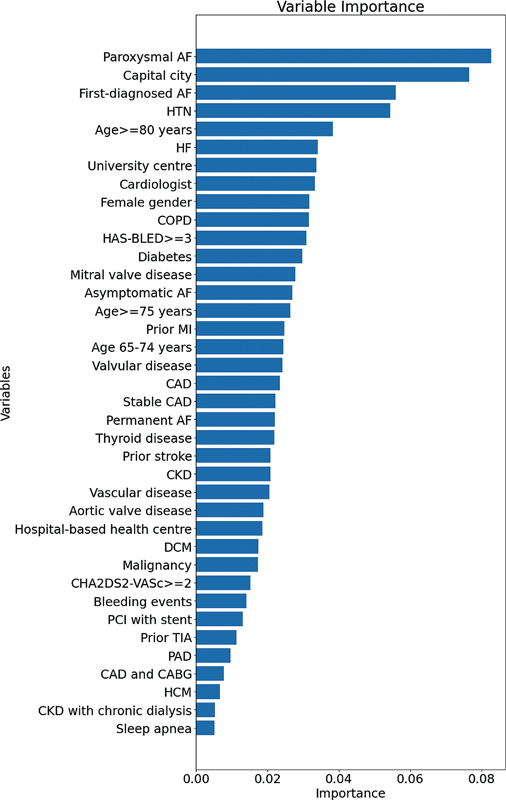
**Variable importance using random forest generator with fine tuning model.** Abbreviations: AF, atrial fibrillation; CABG, coronary artery bypass grafting; CAD, coronary artery disease; CHA _2_ DS _2_ -VASc; HAS-BLED, hypertension, abnormal renal /liver function, stroke, bleeding history or predisposition, labile International Normalised Ratio (INR), elderly (>65 years), drugs or alcohol concomitantly; HCM, hypertrophic cardiomyopathy; HF, heart failure; HTN, hypertension; MI, myocardial infarction; PAD, peripheral artery disease; PCI, percutaneous coronary intervention; TIA, transient ischemic attack.

The most important variables from the random forest generator model were: paroxysmal AF, center in capital city, and first-diagnosed AF, Fig. 2 .

Machine learning evaluated predictors of ‘A-criterion adherent management’ were good (area under the receiver-operator curve = 0.710 (95% CI 0.67–0.75) for random forest with fine tuning.

We were not able to calculate area under the receiver-operator curve for the support vector machine, as this method does not support converting a decision into a probability score, so we could not pass the necessary scores into functions. We compared models using a confusion matrix.

## Discussion

In this ‘proof of concept’ analysis from the BALKAN-AF survey, we provide a novel approach to improve our determination of predictors of adherence with ‘A’ (avoid stroke) component, by using ML algorithms. ML is advancing our understanding of AF in relation to the predictors of adherence with ‘A’ (avoid stroke) component of the ABC pathway. New independent predictors of adherence with ‘A’-criterion of the ABC pathway were identified. Those predictors may help to improve optimization of integrated care in AF patients and filling the knowledge gaps in stroke prevention therapy.

The most important variables from the random forest generator model in decreasing order importance, were paroxysmal AF, center in capital city, and first-diagnosed AF.

The CHA _2_ DS _2_ -VASc score was an independent predictor of ‘A’-criterion adherent management according to our ML algorithms; however, the CHA _2_ DS _2_ -VASc score was not associated with ‘A’-criterion adherent management according to the simple statistical logistic regression model, as previously reported. 11 Indeed, in the Balkan region the overall use of OAC for stroke prevention was poorly associated with individual patient stroke risk. 12 This might be related to local standard of care, reimbursement of medication, ability to access the medication or physician specialty. Importantly, special focus should be given to improvement of anticoagulation use in AF patients in Balkan region.

CHA _2_ DS _2_ -VASc score was that its utility is limited outside of clinical research or the academic setting. 14 These knowledge gaps should be addressed by targeted educational and advocacy efforts, with health economic implications. 15 16

Another independent predictor of ‘A’- criterion adherent management in the Balkan region was HAS-BLED score. This finding implies that either the significance of HAS-BLED score was correctly interpreted or the score might have been ignored. Moreover, stroke risk is closely associated with bleeding risk, and some thromboembolic risk factors like older age, hypertension or history of stroke have also been classified as bleeding risk factors. 17 It should be emphasized that HAS-BLED score should be used to identify modifiable bleeding risk factors and flag up the high risk patients for early review and follow-up. 18 Indeed, the HAS-BLED score when used appropriately is associated with lower major bleeds and an increase in OAC use at 1 year 14 and should not be used as an excuse to withhold OAC. In one study, knowledge gaps associated with HAS-BLED score were reported as needing improvement by 32% of cardiologists. 14

In our study, chronic kidney disease (CKD) on haemodialysis was independently associated with a lower likelihood of ‘A’-criterion adherent management. A similar pattern has been reported from other cohorts. 19 20 Independent of AF, CKD is a pro-haemorrhagic and prothrombotic condition. 21 22 Indeed, patients with CKD and AF are also at higher risk of myocardial infarction, major bleeding, and death. 21

Using ML algorithms, we also found a tendency for OAC to be used more in patients with paroxysmal AF. In contrast to the ML methods, paroxysmal AF was associated with a lower likelihood of A-adherent management based on logistic regression. According to AF guidelines, OAC should be prescribed on the basis of stroke risk factors irrespective of the temporal pattern of AF. 1 23

First-diagnosed AF was associated with higher likelihood of ‘A’-criterion adherent management, and importantly, the decision on OAC use should be based on the presence of conventional stroke risk factors (CHA _2_ DS _2_ -VASc score). 1 In the BALKAN-AF survey, the capital city was associated with increased likelihood of ‘A’-criterion adherent management, consistent with OAC being more commonly prescribed by tertiary care centres than in district hospitals. 24 The importance of awareness in the periphery of Balkan countries should be underlined. Patients with AF are managed by physicians with various types of training, and their perception of AF may affect management decisions, re-emphasizing the need for a common approach to holistic care.

Guideline non-adherence is multifactorial, including healthcare professional/ physician- and healthcare system-related factors. 25 26 Integrated AF care may streamline adherence to guidelines. Hence, education and counselling should be part of any integrated management of patients with AF, to improve their understanding and compliance and adherence to management. 2

Machine-learning is increasingly used in AF research to improve detection, risk analysis and improve AF management. 8 In many instances, such machine learning approaches substantially improve on simple clinical risk models, 27 28 and offer the opportunity to be incorporated into mobile health solutions. 29 In the present analysis, there was good prediction of A-criterion adherence. However, there is a possibility that statistical improvement may not necessarily equate to clinical and practical improvements.

## Limitations

Our study has limitations that should be reported. First, BALKAN-AF registry has no follow-up. Second, data on patient's refusal or preference and contraindications regarding stroke prevention therapies were not collected. Third, we also did not have data on disease severity, disability or frailty, which may explain why OAC was not prescribed to some high risk groups (e.g., post stroke). Lastly, the area under curve ROC of the ML models might have been interferred with a large number of features in the model. The AUC coefficient is calculated for predicted values. Coefficients that describe the training data were higher.

## Conclusion

Machine learning identified paroxysmal AF, treatment center in the capital city, and first-diagnosed AF as predictors of adherence to the A pathway; and hypertrophic cardiomyopathy, chronic kidney disease with chronic dialysis, and sleep apnea as predictors of non adherence.

## What's New?

We tested machine learning (ML) algorithms to determine predictors of adherence with ‘A’ (avoid stroke) component. The most important predictors of adherence with the ‘A’ criterion of the ABC pathway can be identified using ML methodology, with good predictive value.
